# Circulating DNA and frequency of colorectal cancer brain metastases in a presumed high-risk group

**DOI:** 10.1038/s41598-023-45939-x

**Published:** 2023-10-30

**Authors:** Louise Bach Callesen, Anders Kindberg Boysen, Rikke Fredslund Andersen, Rikke Beese Dalby, Karen-Lise Garm Spindler

**Affiliations:** 1 Department of Oncology, Aarhus University Hospital, Palle Juul-Jensens Boulevard 99, 8200 Aarhus N, Denmark; 2 Institute of Clinical Medicine, Aarhus University, Aarhus, Denmark; 3 Department of Biochemistry and Immunology, Vejle Hospital, University Hospital of Southern Denmark, Vejle, Denmark; 4 Department of Radiology, Aarhus University Hospital, Aarhus N, Denmark; 5 Department of Radiology, Esbjerg Hospital, University Hospital of Southern Denmark, Esbjerg, Denmark

**Keywords:** Cancer, Gastrointestinal cancer, Colorectal cancer

## Abstract

This explorative prospective observational pilot study investigated if suggested risk factors, rectal cancer and lung metastases, could add to a relevant detection rate of asymptomatic brain metastases (BM) from colorectal cancer (CRC). Secondary, prognostic biological aspects were investigated by translational analysis of plasma samples. The study enrolled patients with rectal cancer and lung metastases. At inclusion, patients underwent a standard MRI scan of the brain. Cell-free DNA (cfDNA) level was measured by a direct fluorescence assay (DFA), and circulating tumor DNA (ctDNA) by ddPCR. BM was detected in one of twenty-nine included patients. Patients had higher cfDNA levels than healthy subjects (p < 0.01). Patients with the primary tumor in situ had higher cfDNA levels than those with resected primary tumor (p < 0.01). Patients with liver involvement had higher cfDNA levels (p = 0.12) and circulating tumor DNA levels (p = 0.01) than those without liver involvement. In conclusion, the modest incidence of BM does not justify routine MRI of the brain in this selected population. cfDNA by DFA could be a valuable tool when planning treatment and follow-up for CRC patients. Future studies should focus on identifying further characteristics and biomarkers associated with a high risk of BM, enhancing the possibility for early intervention.

## Introduction

Brain metastases (BM) are an uncommon presentation of metastatic colorectal cancer (mCRC), with reported incidence ranging from 0.1 to 11.5%^1,2^. Hence, routine imaging of the brain is not recommended despite BM often being asymptomatic^2^. Patients with BM who undergo a palliative treatment course have an expected survival of a few months^2^. However, intensified surveillance of patients at risk of developing BM could potentially lead to earlier detection and other treatment options. Appropriately selected patients could be candidates for metastasis-directed treatment with a potential for a curative outcome and longer survival^3^. In a registry-based colorectal cancer (CRC) cohort study, patients undergoing curatively intended treatment of BM had more frequent rectal cancers with lung metastases indicating a higher risk of BM in patients with rectal cancer and lung metastasis^3^. Selection criteria to identify patients at risk for developing BM are needed.

In the past decade, the prognostic value of circulating free DNA (cfDNA) and circulating tumor DNA (ctDNA) in patients with mCRC have been heavily investigated^4,5^, but only seldom in relation to BM.

The primary aim of this explorative pilot study was to prospectively investigate if the currently suggested risk factors, rectal cancer and lung metastases, could add to a relevant detection rate of asymptomatic BM from CRC. Secondary to investigate possible prognostic biological aspects by translational analysis of plasma samples.

## Methods

The study was approved by The Central Denmark Region Committees on Health Research Ethics (1-10-72-269-20), and it was prospectively registered with ClinicalTrials.gov (ClinicalTrials.gov identifier: NCT05185557). The research was carried out according to the principles set out in the Declaration of Helsinki 1964 and all subsequent revisions. Written and orally informed consent was obtained from all study participants.

### Study design

The BRAINSTORM study was a prospective single-arm observational pilot study. The study prospectively enrolled patients with rectal cancer and lung metastases.

### Patients

Patients with rectal cancer and lung metastases were included at the Department of Oncology, Aarhus University Hospital (Aarhus, Denmark). The key inclusion criteria were rectal cancer, lung metastasis diagnosed by histo- or cytopathology, or clinical and imaging criteria. The key exclusion criteria were contraindications for magnetic resonance imaging (MRI), previously treated or known BM. Hence, patients were included independent of treatment of the primary tumor and the presence of other metastatic sites. Also, patients in follow-up after loco-regional treatment of lung metastases were allowed inclusion.

For comparison, blood samples were available from 94 fully anonymized presumed healthy individuals (ClinicalTrials.gov identifier: NCT02482896), as previously described^6^. All healthy donors gave written informed consent, and the study was approved by Region Zealand's Ethical Committee on Health Research (approval no. SJ‑421).

### Study procedures

At inclusion, patients underwent a standard 3 T MRI scan of the brain with intravenous contrast. A specialized radiologist described the MRI scan. Positive findings were discussed at the multidisciplinary tumor board for potential treatment options according to best standard of care. All patients underwent follow-up according to standard of care.

### Collection of samples for cell-free DNA analysis

Plasma samples were collected at inclusion. A total of 34 ml of whole blood was drawn. Plasma samples were obtained in EDTA tubes. Plasma was isolated by double centrifugation at 1600×*g* for 10 min and 10,000×*g* for 10 min. The centrifugations were done at room temperature within 2 h and stored at – 80 °C until further analysis.

The levels of total cfDNA in plasma samples were measured by a direct fluorescence assay (DFA). Furthermore, plasma samples were analyzed by ddPCR, measuring methylation of the *NPY* gene. The samples were analyzed blinded to clinical parameters and study endpoints.

### Direct fluorescent assay

A modified version of the method described by Goldshtein et al. detected cfDNA levels directly in the plasma samples^6,7^. Concisely, 40 μl of plasma was added to 160 μl PBS containing DMSO (1:8) and SYBR® Gold Nucleic Acid Gel Stain (1:8000; Invitrogen; Thermo Fisher Scientific, Inc.). Fluorescence was measured with the 96-well fluorometer (Infinite F200 PRO; Tecan Group) at an emission wavelength of 535 nm and an excitation wavelength of 485 nm. Standards for DNA were prepared from Human Control Genomic DNA (Thermo Fisher Scientific, Inc.) diluted in PBS containing 10% bovine serum albumin (Sigma Aldrich; Merck KGaA). The concentration of cfDNA in the plasma samples was calculated from a standard curve, and the final concentration of each sample was calculated as the mean of four measurements. Outliers were removed according to Dixon's q-test if the standard deviation (SD) exceeded 10% if the removal of one value was able to bring the SD < 10%; otherwise, the sample was discarded if SD > 15%.

### Methylation analysis

Tumor-specific methylated DNA was defined as DNA with methylation of the *NPY* gene.

cfDNA was purified from 4 ml of plasma on the QIAsymphony SP instrument using QIAsymphony DSP circulating DNA kit (Qiagen, Hilden, Germany) or the Maxwell purification instrument using the Maxwell RSC ccfDNA LV plasma kit according to the manufacturer’s instructions. Before concentrating the purified DNA, 30 μl of the 200 μl was saved for quality assurance tests. The purified DNA was concentrated to 20 µl on Amicon Ultra Centrifugal filter units (Millipore, Burlington, MA, USA) and was bisulfite converted in a 150 µl reaction with the EZ DNA Methylation lightning spin-column kit (Zymo Research, Irvine, CA, USA) and eluted in 15 µl. The converted DNA was analyzed with a methylation-specific assay and a control assay (Albumin gene) using the BioRad Droplet Digital PCR system QX200 (BioRad, Hercules, CA, USA). The Albumin/*NPY* duplex analysis was performed in two wells with 5 µl converted DNA per well in 20 µl reactions. ddPCR supermix for probes (no Deoxyuridine Triphosphate) and Albumin/*NPY* assays were applied^7^. Droplets were generated on the QX200 automated droplet generator from BioRad. PCR was completed on the Veriti PCR device (Thermo Fisher Scientific, Waltham, MA, USA). The QX200 droplet reader from BioRad counted droplets. Data analysis was performed with QuantaSoft by BioRad version 1.7.4.

Water and a pool of DNA from whole blood from non-cancer individuals were used as negative controls, and universal human methylated control DNA (Zymo Research) and EpiTect control DNA (Qiagen) were used as positive controls. Negative controls and universal human methylated control DNA were bisulfite converted together with the samples.

Three parameters were evaluated for quality assurance of analysis results. Firstly, the potential loss of DNA during purification and analysis was evaluated using a CPP1 assay. Secondly, potential contamination of the purified DNA with DNA from white blood cells was evaluated using an immunoglobulin gene-specific assay (PBC)^8^. Thirdly, to control cfDNA amount and sample fragmentation, an in-house multiplex ddPCR reaction was performed, amplifying a 65 bp and 250 bp fragment of the EMC7 gene 5^9^. All controls were included in each round of ddPCR.

### Statistics

The study was designed as a pilot investigation, with a Simons two-stage design, initially including 29 patients. If two patients were identified with BMs, the study would qualify for continuation into stage two with another 44 patients. A possible dropout of 15% has been taken into account.

A patient is defined as having progressive disease at inclusion, if a CT scan within one month of inclusion showed progressive disease according to RECIST version 1.1 without the patient having started oncological treatment after the scan was performed. Patients are defined as having no evidence of disease at inclusion if there was no visible disease on the latest CT scan before inclusion. All other patients are defined as having stable disease at inclusion.

Categorized variables were expressed as counts and proportions, and continuous variables as mean values, median values, and ranges. The Mann‑Whitney *U*‑test was applied to examine the association between patient characteristics and cfDNA levels. Fisher's exact test was applied to compare categorical variables. We performed analysis of the receiver-operating-characteristic (ROC) curve to evaluate diagnostic performance. The optimal cutoff value was estimated by the Youden method. All reported p‑values were two‑sided, and p < 0.05 was considered to indicate statistical significance. Effect sizes were indicated by 95% confidence intervals (95% CI). Statistical analyses were performed using STATA/IC17.0 (StataCorp LLC).

## Results

**Figure 1:**
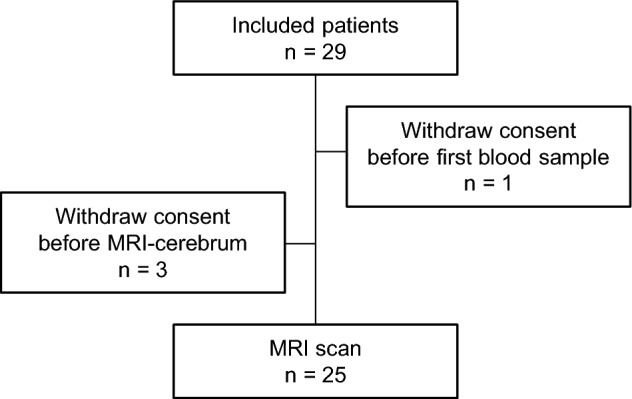
Study flow.

From August 2021 to February 2022, 29 patients were included. Four patients withdrew their consent for further study-specific procedures before the planned study-specific MRI scan. The remaining 25 patients underwent a standard 3 T MRI scan of the brain with intravenous contrast. The study flow is presented in Fig. 1, and patients' characteristics are presented in Table 1.

**Table 1:** Baseline patient characteristics (n = 27) and association with cfDNA levels and detection of ctDNA.

	n (%)^a^	Total cfDNA level by DFA		ctDNA detection by ddPCR		
		ng/µl plasma	p-value^b^	No	Yes	p-value^c^
		Median (95% CI)		n (%)	n (%)	
Age, median (range)	67 (40–90)					
< 67 years	13 (48%)	0.73 (0.58–1.08)	0.55	6 (46%)	7 (54%)	0.71
≥ 67 years	14 (52%)	0.73 (0.60–0.89)		8 (57%)	6 (43%)	
Sex						
Female	10 (37%)	0.73 (0.58–0.97)	0.71	5 (50%)	5 (50%)	1.00
Male	17 (63%)	0.78 (0.60–1.06)		9 (53%)	8 (47%)	
WHO performance status						
0	8 (30%)	0.67 (0.50–0.94)	0.74^d^	6 (75%)	2 (25%)	0.62^d^
1–2	9 (33%)	0.75 (0.56–1.02)		5 (56%)	4 (44%)	
Missing	10 (37%)	0.85 (0.58–1.50)		3 (30%)	7 (70%)	
Primary tumor resected?						
Yes	22 (81%)	0.68 (0.60–0.82)	< 0.01	13 (59%)	9 (41%)	0.17
No	5 (19%)	1.10 (0.78–1.91^e^)		1 (20%)	4 (80%)	
Disease status						
No evidence of disease	5 (19%)	0.62 (0.35–1.04^e^)	0.16^f^	4 (75%)	1 (25%)	0.06
Stable disease	10 (37%)	0.73 (0.60–0.89)	0.35^f^	7 (70%)	3 (30%)	
Progressive disease	12 (44%)	0.81 (0.59–1.09)		3 (25%)	9 (75%)	
Number of metastatic sites						
0	5 (19%)	0.62 (0.35–1.04^e^)	0.35^g^	4 (80%)	1 (20%)	0.02
1	7 (26%)	0.86 (0.58–1.10)	0.53^g^	6 (86%)	1 (14%)	
≥ 2	15 (56%)	0.75 (0.60–0.89)		4 (27%)	11 (73%)	
Liver metastases?						
No	19 (70%)	0.65 (0.59–0.91)	0.12	12 (63%)	7 (37%)	0.10
Yes	8 (30%)	0.81 (0.67–1.77)		2 (25%)	6 (75%)	
Mutational status in tumor tissue						
Wild-type	9 (33%)	0.73 (0.56–1.06)	0.78^d^	5 (56%)	4 (44%)	1.00^d^
Mutated	14 (52%)	0.69 (0.60–0.83)		7 (50%)	7 (50%)	
Missing	4 (15%)	0.91 (0.72–1.10^e^)		2 (50%)	2 (50%)	

*WHO* World Health Organization, *cfDNA* cell-free DNA.

^a^Percentage may not total 100 due to rounding.

^b^Mann–Whitney *U*-test.

^c^Fisher’s exact test.

^d^Comparison not including patient with missing values.

^e^Upper confidence limit held at maximum of sample.

^f^Compared to 'progressive disease'.

^g^Compared to 'Number of metastatic sites' ≥ 2.

Evidence of BM was detected in one patient (4.0%). Consequently, the study was not continued into the next phase, but inclusion stopped.

### Patient characteristics

For the 27 patients with available baseline plasma samples, baseline patient characteristics are presented in Table 1. The median age was 67 years (range 40–90 years), and the majority were male (63%). Twenty-two patients had active lung metastases, including seven with lung-only disease, whereas five patients were included during follow-up after loco-regional treatment of lung metastases. Mutational status in tumor tissue comprised 14 patients (67%) with *KRAS* mutations, nine without detection of *KRAS*, *NRAS*, or *BRAF* mutations (wild-type), and four without available mutational testing.

### Total circulating free DNA

**Figure 2:**
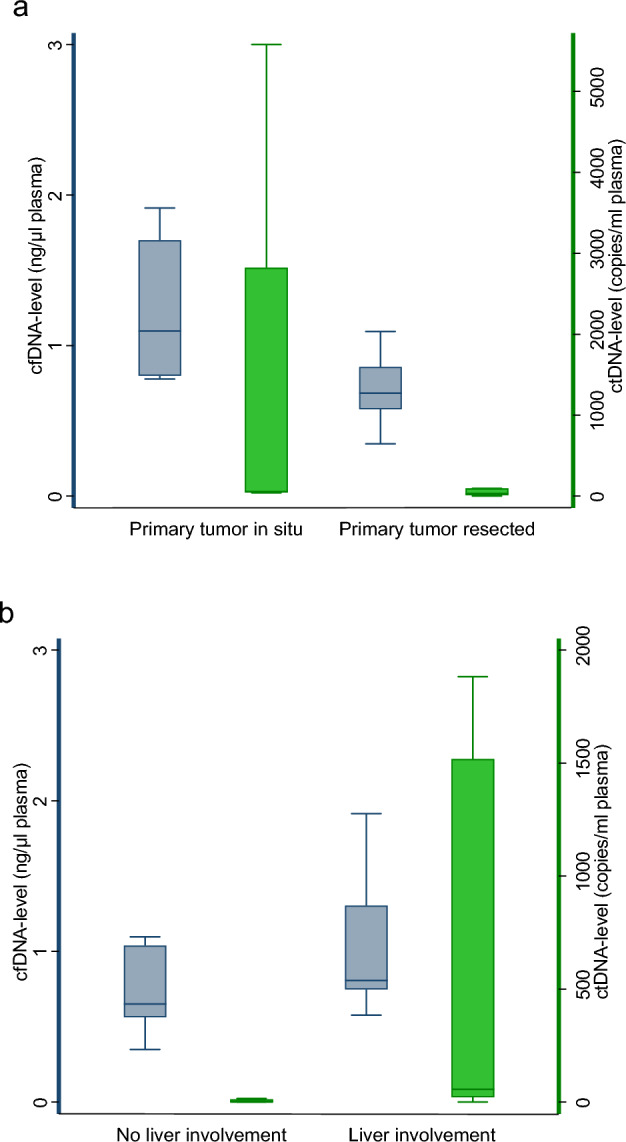
Cf- and ctDNA levels and patient characteristics. Association of baseline plasma cfDNA levels (n = 27) and ctDNA levels (n = 13) with primary tumor status (**a**) and liver involvement (**b**) in patients with lung metastases from rectum cancer. Box and whisker plot with 25th, 50th, and 75th percentiles, upper and lower adjacent values of cfDNA levels. Outliers not shown. cfDNA, cell‑free DNA; ctDNA, circulating tumor DNA.

The total cfDNA levels were measured by DFA in all available baseline plasma samples (n = 27) with a median value of 0.73 ng/µl plasma, a mean value of 0.81 ng/µl plasma, and a range of 0.35–1.91 ng/µl plasma. We report significantly higher cfDNA levels in patients with the primary tumor in situ (median = 1.10 ng/µl plasma, 95% CI 0.78–1.91 ng/µl plasma) compared to patients with resected primary tumor (median = 0.68 ng/ml plasma, 95% CI 0.60–0.82 ng/µl plasma, p < 0.01; Fig. 2A). Furthermore, we report a tendency to higher cfDNA levels in patients with liver metastases (median = 0.81 ng/µl plasma, 95% CI 0.67–1.77 ng/µl plasma) compared to patients without liver involvement (median = 0.65 ng/µl plasma, 95% CI 0.59–0.91 ng/µl plasma, p = 0.12; Fig. 2B) (Table 1).

**Figure 3:**
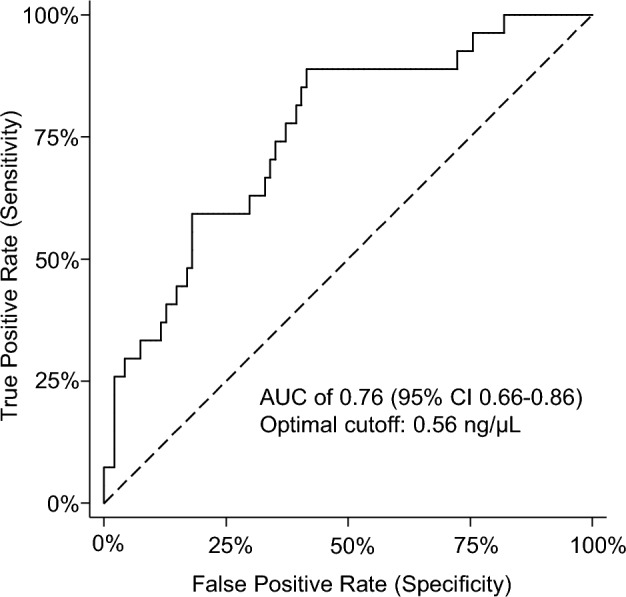
Receiver Operating Characteristic of baseline plasma cfDNA levels between the healthy cohort and the studied cohort. The AUC was 0.76 (95% CI 0.66–0.86). The optimal cut-off was estimated to 0.56 ng/μL with a sensitivity of 89% and a specificity of 59%.

Baseline cfDNA levels in the studied cohort were compared to cfDNA levels of a cohort of healthy individuals. In the cohort of 94 healthy individuals, the total cfDNA levels were measured by DFA with a median value of 0.52 ng/µl plasma, a mean value of 0.54 ng/µl plasma, and a range of 0.13–1.47 ng/µl plasma. Significantly higher cfDNA levels were observed in the studied cohort compared to the healthy individuals (p < 0.001). A ROC analysis was applied with an AUC of 0.76 (95% CI 0.66–0.86) and an optimal cutoff of 0.56 ng/μL with a sensitivity of 89% and specificity of 59% for a distinction between healthy subjects and patients in the study cohort (Fig. 3).

### Circulating tumor DNA

All available baseline plasma samples were analyzed for the presence of ctDNA, defined as the detection of DNA with methylation of the *NPY* gene. In total, ctDNA was detected in 13/27 baseline plasma samples (48%). With more advanced disease, a higher fraction of patients had ctDNA detected. Only 14% of patients with one metastatic site had detectable ctDNA, whereas 73% of patients with two or more metastatic sites had detectable ctDNA. Of note, one patient with no evidence of disease had detectable ctDNA. This patient was diagnosed with relapse less than a year after inclusion (Table 1).

The median level of ctDNA in the ctDNA-positive patients was 48.8 copies/ml plasma (range: 1.8–5578.2 copies/ml plasma, n = 13). The median fraction of ctDNA was 5.2% (range: 0.04%-45.1%, n = 13). Furthermore, we report significantly higher ctDNA levels in patients with liver metastases (median = 608.3 copies/ml plasma, 95% CI 42.7–5208.6 copies/ml plasma) compared to patients without liver involvement (median = 16.5 copies/ml plasma, 95% CI 4.9–81.8 copies/ml plasma, p = 0.01; Fig. 2B).

## Discussion

In this presumed high-risk population, we only detected BM in one patient; consequently, we did not continue the inclusion of patients. However, in this study, we allowed both inclusions of patients with active disease and patients having undergone localized treatment for lung metastases to allow for assessment of the potential situation of oligometastatic BM. In conclusion, our selection criteria were insufficient to identify a relevant risk group. The observed case with identified asymptomatic BM was a patient with active lung metastases.

Earlier detection of BM may lead to better treatment options, including metastasis-directed treatment with a potential for a curative outcome leading to a higher quality of life and prolonged survival.

We suggest that further selection criteria are needed e.g. HER2 amplification, positive level of CEA, pN2a-b, and distant metastases^10,11^. Circulating DNA has proven a promising tool for identifying minimal residual disease and early failure detection in this disease^12^. Studies suggest a clinically relevant potential lead time between clinically detected recurrence and the molecular biological detected disease in liquid biopsies^13^. In the widespread setting, circulating tumor DNA also holds important clinical implications. ctDNA analyses can aid in the treatment selection, detection of treatment response and treatment failure, and as a prognostic marker^5^. However, little is known about the shedding of cfDNA from BM, and whereas the present sample size is inadequate to evaluate this specific topic, we present clinically relevant observations of the combined measurement of cfDNA and ctDNA detected by methylation of the *NPY* gene.

In our study, we used a novel and pragmatic approach to circulating DNA analysis, i.e., a tumor-agnostic ctDNA platform and a simple total cfDNA measurement. A tumor-agnostic detection of ctDNA in plasma is valuable since this overcomes the need for tumor biopsies and heterogeneity. Methylation of the *NPY* gene is a well-known universal marker of ctDNA in patients with CRC and was used in our pilot investigation^14^. In the present study, we detected ctDNA in 12 of 22 patients with radiological evident disease, with the highest detection frequency among patients with widespread disease (≥ 2 metastatic sites), indicating that patients with widespread disease may have a higher level of ctDNA. We observed a tendency for a higher frequency of ctDNA detection in patients with liver metastases, suggesting that liver metastases have a higher shedding of ctDNA compared to metastases in other locations, e.g., lung metastases, which is in accordance with the existing literature^15–17^.

In addition to ctDNA, it is reported that total cfDNA levels hold prognostic value in patients with mCRC^18^. A fast, easy, low-cost laboratory method analyzing cfDNA is attractive. DFA is a new in-house developed method for cfDNA measurement directly in plasma. For validation of this new, promising method, cfDNA measurements in the study cohort were compared with a cohort of healthy individuals. In accordance with the existing literature, we report significantly higher cfDNA levels in the study cohort compared to a presumed healthy cohort^19,20^. With the optimal cutoff of 0.56 ng/μl, the cfDNA level was able to discriminate between patients in the study cohort and healthy individuals with a sensitivity of 89% and specificity of 59%.

Furthermore, in accordance with the few published data, we found higher cfDNA levels among patients with the primary tumor in situ and in patients with liver metastases^20–23^. This observation again points in the direction that the shedding of ctDNA is higher in liver tissue than in other locations, e.g., lung tissue. In conclusion, circulating DNA levels and tumor DNA detection seem correlated to more advanced disease and the presence of liver metastases. Due to the low detection rate of BM, it is not possible to evaluate if circulating DNA could be relevant, when identifying patients with a risk of BM from CRC. Further translational research is needed.

The present study was a pilot study with obvious limitations, including the relatively low sample size and the low frequency of BM hindering the evaluation of possible prognostic biological aspects in patients with BM from CRC by translational analysis of plasma samples. Future studies in more homogenous study cohorts and further translational research are needed to identify patients with a high risk of BM from this disease.

In conclusion, a single asymptomatic BM was detected, but we did not find an incidence of BM, which justifies routine MRI of the brain of all patients in this selected population. Circulating DNA measured by DFA, could be a valuable tool when planning treatment and follow-up for patients with CRC. Future studies should focus on identifying further characteristics and biomarkers associated with high risk of BM from CRC. This would enable early detection of BM and thereby a possibility for early intervention, prolonged survival, and improved quality of life.

## Data Availability

The datasets used and/or analyzed during the current study are available from the corresponding author upon reasonable request.
